# Supplementary material for: Statistical refinement of patient-centered case vignettes for digital health research
Source: Front Digit Health. 2024 Oct 21;6:1411924. doi: 10.3389/fdgth.2024.1411924 (PMC11532084; doi:10.3389/fdgth.2024.1411924)
Supplement: Supplementary file 1 [file Table1.docx]

**Supplementary Table 1.** Refined vignette set for laypeople.

| Symptom cluster | Case number | Link to case | Assigned urgency level |
| --- | --- | --- | --- |
| Musculoskeletal Pain | 1 | <https://www.reddit.com/r/AskDocs/comments/14enray/siderib_injury/> | Self-care |
|  | 2 | <https://www.reddit.com/r/AskDocs/comments/14fshtx/shin_pain_after_run_28f/> | Self-care |
|  | 3 | <https://www.reddit.com/r/AskDocs/comments/14i0dv6/16m_arm_hurt_when_curl_heavy_weight/> | Self-care |
| Joint Pain | 4 | <https://www.reddit.com/r/AskDocs/comments/14gywqb/radiating_pain_in_leg_after_rolling_ankle_30m/> | Self-care |
|  | 5 | <https://www.reddit.com/r/AskDocs/comments/14hh03i/fell_on_my_hands_and_thumb_hurts/> | 1-Day-Urgent |
| Headache | 6 | <https://www.reddit.com/r/AskDocs/comments/14j1dou/really_bad_anxiety_after_hitting_head/> | Emergency |
| Other Pain | 7 | <https://www.reddit.com/r/AskDocs/comments/14ckwcp/electric_shock_in_neck_and_armpit/> | Non-Emergency |
|  | 8 | <https://www.reddit.com/r/AskDocs/comments/14ggsg8/kidney_infection/> | 1-Day-Urgent |
|  | 9 | <https://www.reddit.com/r/AskDocs/comments/14ay33z/dull_ache_in_left_testicle/> | Non-Emergency |
| Gynecological | 10 | <https://www.reddit.com/r/AskDocs/comments/14ft7ys/is_it_possible_to_have_yeast_infection_only_on/> | Non-Emergency |
|  | 11 | <https://www.reddit.com/r/AskDocs/comments/14g6ob6/period_for_25_weeks/> | 1-Day-Urgent |
|  | 12 | <https://www.reddit.com/r/AskDocs/comments/14gsz4w/no_period_in_4_months/> | Non-Emergency |
| Tumors/lumps/masses | 13 | <https://www.reddit.com/r/AskDocs/comments/14k8uv2/i_just_found_this_random_scar_and_lump_in_the/> | Self-care |
|  | 14 | <https://www.reddit.com/r/AskDocs/comments/14mchud/calf_knots/> | Non-Emergency |
| Edema | 15 | <https://www.reddit.com/r/AskDocs/comments/14jzlhh/why_do_my_legs_do_this/> | Non-Emergency |
| Skin issues | 16 | <https://www.reddit.com/r/AskDocs/comments/14ltfn7/skin_changes_on_my_thighs_photos_on_comments/> | Non-Emergency |
| Gastrointestinal | 17 | <https://www.reddit.com/r/AskDocs/comments/14l2p0e/can_someone_help_me/> | Self-care |
| Impaired sensations | 18 | <https://www.reddit.com/r/AskDocs/comments/14ixbzd/19m_tingling_sensation_in_hands_and_feet_during/> | Self-care |
|  | 19 | <https://www.reddit.com/r/AskDocs/comments/14l0gge/wrist_issue/> | Self-care |
| Urinary Tract Problems | 20 | <https://www.reddit.com/r/AskDocs/comments/14jx77b/uti_or_friction/> | Non-Emergency |
|  | 21 | <https://www.reddit.com/r/AskDocs/comments/14lq0oj/weird_tingly_pressure_in_bladder_constant_urge_to/> | 1-Day Urgent |
| Chest Pain and Upper Respiratory Symptoms | 22 | <https://www.reddit.com/r/AskDocs/comments/14i5y0o/105_fever_and_sore_abslower_belly/> | Self-care |
| Other | 23 | <https://www.reddit.com/r/AskDocs/comments/14l9bpt/blood_in_my_stool/> | Non-Emergency |
|  | 24 | <https://www.reddit.com/r/AskDocs/comments/14hynsg/dog_tooth_scraped_cartridge_on_inside_of_my/> | 1-Day Urgent |
|  | 25 | <https://www.reddit.com/r/AskDocs/comments/14ckxmz/should_i_go_to_the_er/> | Emergency |
|  | 26 | <https://www.reddit.com/r/AskDocs/comments/14jbuuu/i_inhaled_a_macaroni_noodle_and_am_scared_of/> | Self-care |
|  | 27 | <https://www.reddit.com/r/AskDocs/comments/14ly5jh/popping_lung/> | Self-care |

**Supplementary Table 2.** Refined vignette set for SAAs.

| Symptom cluster | Case number | Link to case | Assigned urgency level |
| --- | --- | --- | --- |
| Musculoskeletal Pain | 1 | <https://www.reddit.com/r/AskDocs/comments/14fshtx/shin_pain_after_run_28f/> | Self-care |
|  | 2 | <https://www.reddit.com/r/AskDocs/comments/14i0dv6/16m_arm_hurt_when_curl_heavy_weight/> | Self-care |
|  | 3 | <https://www.reddit.com/r/AskDocs/comments/14kxdjj/swollen_foot_no_clot/> | Non-Emergency |
| Joint Pain | 4 | <https://www.reddit.com/r/AskDocs/comments/14avegt/i_cant_identify_this_shoulder_pain_please_help/> | Non-Emergency |
|  | 5 | <https://www.reddit.com/r/AskDocs/comments/14hh03i/fell_on_my_hands_and_thumb_hurts/> | 1-Day-Urgent |
| Chest Pain | 6 | <https://www.reddit.com/r/AskDocs/comments/14epjg6/lungs_pain_inside_lung_pain/> | 1-Day-Urgent |
| Other Pain | 7 | <https://www.reddit.com/r/AskDocs/comments/14ckwcp/electric_shock_in_neck_and_armpit/> | Non-Emergency |
|  | 8 | <https://www.reddit.com/r/AskDocs/comments/14ggsg8/kidney_infection/> | 1-Day-Urgent |
|  | 9 | <https://www.reddit.com/r/AskDocs/comments/14ay33z/dull_ache_in_left_testicle/> | Non-Emergency |
| Gynecological | 10 | <https://www.reddit.com/r/AskDocs/comments/14ft7ys/is_it_possible_to_have_yeast_infection_only_on/> | Non-Emergency |
|  | 11 | <https://www.reddit.com/r/AskDocs/comments/14g6ob6/period_for_25_weeks/> | 1-Day-Urgent |
|  | 12 | <https://www.reddit.com/r/AskDocs/comments/14gsz4w/no_period_in_4_months/> | Non-Emergency |
| Tumors/lumps/masses | 13 | <https://www.reddit.com/r/AskDocs/comments/14k8uv2/i_just_found_this_random_scar_and_lump_in_the/> | Self-care |
|  | 14 | <https://www.reddit.com/r/AskDocs/comments/14mchud/calf_knots/> | Non-Emergency |
| Edema | 15 | <https://www.reddit.com/r/AskDocs/comments/14gprss/my_leg_is_a_bit_swollen/> | 1-Day-urgent |
| Skin issues | 16 | <https://www.reddit.com/r/AskDocs/comments/14ltfn7/skin_changes_on_my_thighs_photos_on_comments/> | Non-Emergency |
| Gastrointestinal | 17 | <https://www.reddit.com/r/AskDocs/comments/14ivqxw/diahrea_for_6_weeks/> | Non-Emergency |
| Impaired sensations | 18 | <https://www.reddit.com/r/AskDocs/comments/14l11z8/left_side_of_the_lip_is_numb_like_im_at_the/> | Self-care |
|  | 19 | <https://www.reddit.com/r/AskDocs/comments/14ixbzd/19m_tingling_sensation_in_hands_and_feet_during/> | Self-care |
| Urinary Tract Problems | 20 | <https://www.reddit.com/r/AskDocs/comments/14jx77b/uti_or_friction/> | Non-Emergency |
|  | 21 | <https://www.reddit.com/r/AskDocs/comments/14e00ng/burning_pain_when_peeing_no_way_this_is_an_std/> | 1-Day Urgent |
| Headache and Upper Respiratory Symptoms | 22 | <https://www.reddit.com/r/AskDocs/comments/14i5y0o/105_fever_and_sore_abslower_belly/> | Self-care |
| Other | 23 | <https://www.reddit.com/r/AskDocs/comments/14hynsg/dog_tooth_scraped_cartridge_on_inside_of_my/> | 1-Day Urgent |
|  | 24 | <https://www.reddit.com/r/AskDocs/comments/14ckxmz/should_i_go_to_the_er/> | Emergency |
|  | 25 | <https://www.reddit.com/r/AskDocs/comments/14jmp9t/weird_drop_on_heart_rhythm/> | Non-Emergency |
|  | 26 | <https://www.reddit.com/r/AskDocs/comments/14jbuuu/i_inhaled_a_macaroni_noodle_and_am_scared_of/> | Self-care |
|  | 27 | <https://www.reddit.com/r/AskDocs/comments/14ly5jh/popping_lung/> | Self-care |

The full-text vignettes are available from the authors upon request.
